# Photoimmunotherapy of Ovarian Cancer: A Unique Niche in the Management of Advanced Disease

**DOI:** 10.3390/cancers11121887

**Published:** 2019-11-27

**Authors:** Shubhankar Nath, Mohammad Ahsan Saad, Michael Pigula, Joseph W.R. Swain, Tayyaba Hasan

**Affiliations:** Wellman Center for Photomedicine, Massachusetts General Hospital, Harvard Medical School, Boston, MA 02114, USA; snath.vet2000@gmail.com (S.N.); msaad1@mgh.harvard.edu (M.A.S.); m.pigula94@gmail.com (M.P.);

**Keywords:** Ovarian cancer, targeted therapy, photodynamic therapy, photoimmunoconjugates, photoimmunotherapy, EGFR

## Abstract

Ovarian cancer (OvCa) is the leading cause of gynecological cancer-related deaths in the United States, with five-year survival rates of 15–20% for stage III cancers and 5% for stage IV cancers. The standard of care for advanced OvCa involves surgical debulking of disseminated disease in the peritoneum followed by chemotherapy. Despite advances in treatment efficacy, the prognosis for advanced stage OvCa patients remains poor and the emergence of chemoresistant disease localized to the peritoneum is the primary cause of death. Therefore, a complementary modality that is agnostic to typical chemo- and radio-resistance mechanisms is urgently needed. Photodynamic therapy (PDT), a photochemistry-based process, is an ideal complement to standard treatments for residual disease. The confinement of the disease in the peritoneal cavity makes it amenable for regionally localized treatment with PDT. PDT involves photochemical generation of cytotoxic reactive molecular species (RMS) by non-toxic photosensitizers (PSs) following exposure to non-harmful visible light, leading to localized cell death. However, due to the complex topology of sensitive organs in the peritoneum, diffuse intra-abdominal PDT induces dose-limiting toxicities due to non-selective accumulation of PSs in both healthy and diseased tissue. In an effort to achieve selective damage to tumorous nodules, targeted PS formulations have shown promise to make PDT a feasible treatment modality in this setting. This targeted strategy involves chemical conjugation of PSs to antibodies, referred to as photoimmunoconjugates (PICs), to target OvCa specific molecular markers leading to enhanced therapeutic outcomes while reducing off-target toxicity. In light of promising results of pilot clinical studies and recent preclinical advances, this review provides the rationale and methodologies for PIC-based PDT, or photo-immunotherapy (PIT), in the context of OvCa management.

## 1. Introduction

Ovarian carcinoma (OvCa) is the leading cause of death from gynecological cancers and is the fifth most frequent cause of cancer-related deaths among women in the United States. It is estimated that in 2019, there will be 22,530 new cases of OvCa and 13,980 deaths in the US (www.cancer.org). The overall five-year survival of ~45% has not improved significantly in the past few decades due to the presence of advanced disease at the time of diagnosis and acquired resistance to the spectrum of currently available chemotherapeutic agents [1,2]. OvCa is especially dangerous as the disease can invade neighboring organs in the abdominal cavity and can enter the bloodstream and lymphatic system to form distant metastases. Making matters worse, early-stage disease is often asymptomatic and misdiagnosed as less deadly digestive issues, accounting for late-diagnosis in its advanced stages. About 80% of the patients are diagnosed at an advanced stage after the cancer has spread throughout the peritoneal cavity. These patients are typically treated with aggressive surgical resection followed by chemotherapy [3]. A number of chemotherapeutic regimens exist for OvCa, and the reader is referred to excellent reviews on the current state of chemotherapy regimens [4,5,6,7]. However, even among patients with negative follow up exams, 50% of patients later present with incurable radio- and chemo-resistant disease.

**Figure 1:**
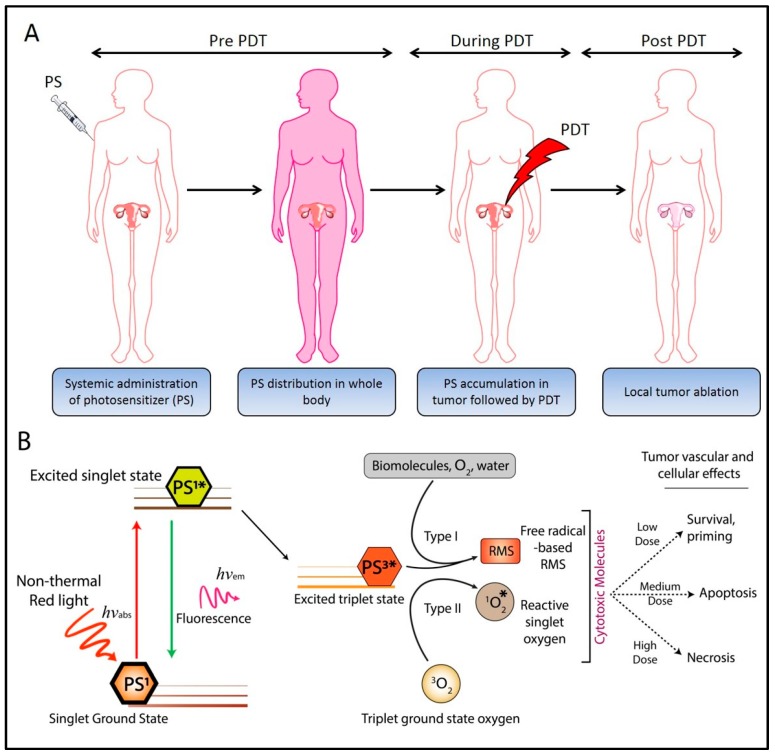
Photobiological and photochemical overview of photodynamic therapy (PDT). (**A**) Process of clinical PDT; the photosensitizer (PS) is administered systemically, followed by an appropriate PS-light interval, where the administered PS preferentially accumulates at the tumor site. Near-infrared (NIR) irradiation of the target tissue leads to localized tumor destruction. (**B**) Schematic representation of the Jablonski diagram showing the ground state of the PS (PS^1^) and the subsequent shift to a high energy excited state (PS^1*^) upon NIR irradiation. The PS in the excited state (PS^1*^) can either emit energy in the form of fluorescence radiation and relax to the ground state or undergo intersystem crossing to generate a long-lived excited triplet state (PS^3*^). Energy and electron transfer from the excited triplet state (PS^3*^) to biomolecules, water, triplet ground state oxygen, etc. leads to the formation of cytotoxic reactive molecular species (RMS). Depending on the dose of the RMS, the target tissues may either survive or undergo apoptosis/necrosis. Adapted from Nath et al. (2019) [26].

Photodynamic therapy (PDT) is a therapeutic and diagnostic modality utilizing the photochemical properties of small molecule photosensitizers (PS). PDT is particularly beneficial for cancer treatment as it imparts two degrees of selectivity—(i) certain formulations of PS preferentially accumulate in cancerous lesions, and (ii) the phototoxicity is limited to regions of tissue irradiated during treatment (Figure 1A). PDT is an FDA-approved anti-cancer treatment modality that has been investigated in preclinical and clinical settings for the management of ovarian [8,9,10,11,12,13,14,15,16,17,18,19,20] and other cancers [21,22]. It is clinically approved in the US for the treatment of numerous cancerous (esophageal and lung cancer) and non-cancerous indications.

PDT has shown particular promise in treating OvCa in preclinical studies [15,20] and clinical trials. Although phase I and II trials in OvCa and other intraperitoneal cancers demonstrated promising results, significant dose-limiting toxicities were also observed [8,9,11]. Major complications included cutaneous phototoxicity and bowel perforation due to non-specific localization of the PS and inadequate light dosimetry. These trials highlighted the clinical potential of PDT in prolonging disease-free survival, with tissue selectivity being a major challenge. In this context, encouraging reports were published by Schmidt et al. [23,24], wherein photoimmunoconjugates (PICs, discussed in Section 3), prepared by the conjugation of phthalocyanine to monoclonal antibodies (MABs) recognizing CA125, were shown to be comparatively more effective in pre-clinical and clinical studies. Since these early studies, considerable advances have been made in PIC-based PDT, or photo-immunotherapy (PIT, discussed in Section 3), suggesting the potential of PIT in the targeted therapy of OvCa. The high degree of spatial and temporal control of cytotoxicity afforded by PIT and its unique mechanism of cell killing make it an ideal candidate for treating regionally localized and resistant disease often seen in OvCa patients. The high recurrence rate even among patients with negative second-look laparotomies is partly attributed to micrometastatic tumor nodules in the peritoneum that are invisible to the eye [25]. Strategies to address these deadly pockets of disease utilizing mechanistically distinct combination therapies that exploit non-overlapping molecular targets represent a promising direction of research in PIT-based treatment of OvCa. PIT has demonstrated promise in overcoming the challenges associated with this deadly disease, and this review will give an overview of preclinical and clinical developments of this therapy and perspective of PIT’s potential role in treating OvCa. 

## 2. Photodynamic Therapy: Mechanisms

PDT is based on the observation that certain formulations of PSs accumulate preferentially in malignant tissues [27,28,29]. Irradiation by a specific wavelength of light leads to the shift in the energy state of the PS from the ground state (PS^1^) to a singlet excited state (PS^1*^). Subsequent intersystem crossing leads to the generation of a long-lived excited triplet state (PS^3*^) from which energy transfer to ground state triplet oxygen leads to the formation of a cytotoxic singlet oxygen species through what is commonly referred to as Type II reactions. Alternatively, the PS in the excited triplet state (PS^3*^) can transfer electrons to biomolecules, water, oxygen, etc., resulting in the formation of a broad variety of additional reactive molecular species (RMS), including radicals and radical ions through what are known as Type I reactions. Depending on the dose of locally generated RMS, the target tissues may either survive or undergo death pathways (Figure 1B). PDT can induce cell death by triggering four different death pathways—apoptosis, necrosis, autophagy, and paraptosis [30,31,32,33]. These pathways can take place concurrently and often depend on PS formulation, intracellular localization [34], PDT dose [35], and cell type. For the scope of this review, we will focus on the mechanisms of PIT-induced apoptotic pathways, which are well documented to induce cancer cell death following PIT.

**Figure 2:**
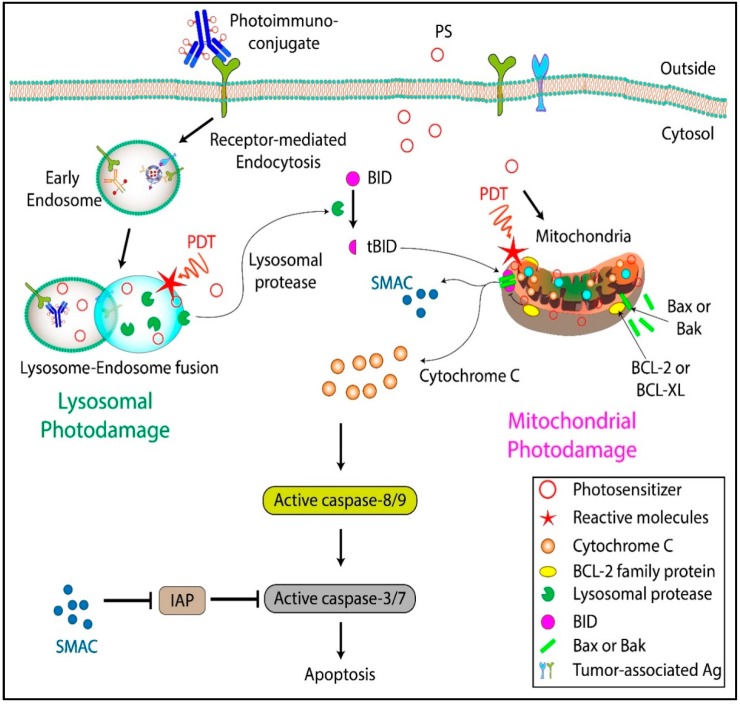
Mechanisms of cell death induced by free PS and photoimmunoconjugates (PICs). PICs bind to the corresponding receptor on the cell surface and are internalized through a receptor-mediated endocytosis pathway. The endocytosed PICs are sorted to the lysosome where they are cleaved, and the PS is released. Certain PSs diffuse through the cell membrane and localize to subcellular organelles, such as mitochondria. Depending on the PS formulation, the cells may undergo either lysosomal photodamage or mitochondrial photodamage leading to apoptosis/necrosis. Adapted from Nath et al. (2019) [26].

**Figure 3:**
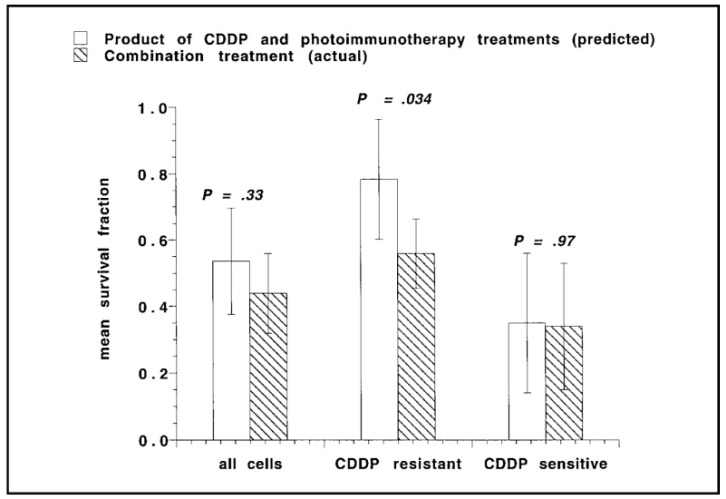
Comparison of predicted and actual mean survival fractions from cells treated with a combination of PIT and cisplatin (CDDP). CDDP-resistant cells were more responsive to combination treatment as compared to CDDP-sensitive cells. Adapted from Duska et al., 1999 [15].

The preferential accumulation sites of PSs are the lysosomes, mitochondria, endoplasmic reticulum (ER), Golgi complex (GC), and the plasma membrane. Plasma membrane–localizing PSs induce cell death mainly through necrosis and membrane rupture [36,37]. In contrast, ER and GC localizing PSs induce cell death by ER stress and inhibition of GC-based secretion of proteins, respectively. PDT with GC localizing PSs has also been shown to induce apoptosis via the activation of caspases [38,39]. PSs that localize in the mitochondrial membranes, among other intracellular membranes, cause cell death by a fast-acting apoptotic mechanism [34,40]. This has been linked to the cytoplasmic release of cytochrome c, which, together with dATP and Apaf-1, leads to the activation of caspase 9 (Figure 2). An important attribute of PDT-induced apoptosis is that the trigger is further downstream in the apoptotic cascade, thus escaping the regulatory effects of proteins such as Bcl-2, overexpression of which is often associated with resistance to chemotherapeutic agents. Interestingly, direct destruction of anti-apoptotic proteins such as Bcl-2 and Bcl-XL by PDT has been demonstrated to result in an altered ratio of pro- and anti-apoptotic factors on the mitochondrial membrane, leading to increased apoptosis and chemotherapy sensitivity in many cancer types [41,42]. Furthermore, PDT can inhibit the activity of P-glycoprotein ATPase and increase the cellular uptake of multidrug resistance (MDR) substrates [43]. Because radical or singlet oxygen species are involved in PDT-based cytotoxicity, many of the major radio- and chemo-resistance mechanisms employed by cancer cells do not impact PDT efficacy. Several OvCa cell lines and tissue samples obtained from patients resistant to radio- and chemo-therapy were shown to be responsive to PIT in studies by Goff et al. [20] and Duska et al. [15] (discussed further in Section 7). The study by Duska et al. highlighted the therapeutic potential and selectivity of PIT in chemoresistant tumors, and the significantly higher efficacy that could be achieved with a combination of PDT and platinum-based chemotherapy in resistant cells, possibly due to mechanistically distinct cell death pathways that were activated by the therapies (Figure 3).

PIC enters cells through receptor-mediated endocytosis and localizes in the lysosomes. Although lysosomes are less photosensitive compared to other sub-cellular organelles [44,45], the selectivity conferred by EGFR targeting of PIC is significant. Moreover, the limitations in PS toxicity can be addressed by increasing the light dose. For lysosomal PDT, the mode of cell death is multifaceted. One pathway is necrosis, possibly due to spillage of lysosomal enzymes and other toxic moieties, or the subsequent PS re-localization to the mitochondria, where they are considerably more phototoxic [46]. Lysosomal PDT also initiates a release of calcium ions leading to calpain-mediated degradation of the autophagy-related protein ATG5, thereby inhibiting autophagy. Lysosomal PDT may also lead to a release of cathepsins that cleave Bid to its truncated form t-Bid, which upon localization to the mitochondria leads to apoptosis (Figure 2). Simultaneous localization and photodamage of multiple subcellular compartments have been shown to increase PDT efficiency [47,48,49], likely due to the induction of cell death through non-overlapping pathways and inhibition of survival mechanisms. Most studies describing PDT that simultaneously targets different organelles utilized two PSs with different localization properties and activation wavelengths. In more recent work, simultaneous targeting of different subcellular organelles with the same PS was reported in 2D and 3D models of OvCa. In these studies, administration of a mixture of liposomal formulations containing free and lipid-anchored BPD was shown to simultaneously target multiple subcellular components and achieve cytotoxic effects at significantly lower light doses [47,49,50]. 

PIT of OvCa and other cancers has been performed using many sensitizers, including chlorins, porphyrins, m-THPC, and phthalocyanines, which commonly follow the typical receptor-mediated endocytosis mechanism. A PIT study with an NIR absorbing phthalocyanine suggested that a photo-induced ligand release mechanism was operative leading to death in OvCa cells [51]. However, given a six-hour incubation period prior to illumination, it is not clear to what extent external ligand cleavage contributed to cell death. Whether part or whole of the cytotoxic effect comes from this ligand cleavage externally or is from the antibody internalization and ligand cleavage in lysosomes, as is the case with most antibody conjugates, was not established. Therefore, the claim that PIT, in this case, is largely from membrane action is not well justified and can only be viewed as a suggestion. The mechanism involved is likely typical receptor-mediated internalization, cleavage and photodynamic action. A number of conventional PSs, particularly photofrin (PF) and other membrane-localizing agents, also aid in cell permeabilization and drug delivery, as shown originally by Henderson et al. and others [52,53,54]. Photochemically, this formulation appears to follow conventional NIR-mediated ROS generation mechanisms, and diminished PIT efficacy was observed in the presence of singlet oxygen scavengers [55]. In the report by Sato et al., the authors add interesting speculation about conformational changes in the antibody post illumination [56]. It would be revealing to see some structural evidence of this either by electron microscopy or NMR, as in the detailed study by Vrouenraets et al. [57], although this would be challenging to investigate in cells.

Apart from the apoptosis/necrosis induced by PDT, many other molecular changes have been documented in cells undergoing PDT. These range from activation of transcription factors [58,59], stimulation of protein kinases [60], expression of stress proteins [61,62], production of cytokines [63,64,65] and prostaglandins [52,66], and oxidation of lipids [67]. 

The mechanisms of tumor destruction in vivo after PDT are equally complex and vary depending on the molecular characteristics of the PS, the route of administration, and the time after administration at which illumination is performed [68]. At the physiological level, there are three main contributing pathways to tumor destruction: (a) Direct tumor cell death [69], thought to be caused by the PS being taken up by tumor cells which then undergo apoptotic [70] or necrotic cell death in a manner analogous to that described in vitro. (b) Vascular effects, where tumor microvessels suffer thrombosis, leakage, and stasis [71], leading to deprivation of oxygen and nutrients, and tumor death by hemorrhagic necrosis [72]. In the context of OvCa, the vascular effects may be less important when the PS is delivered by an intracavitary route and when the primary (but not exclusive) targets are intracellular. Moreover, vascular effects may not be very pronounced if the drug to light interval (DLI) is long, due to diffusion of the PS from the vasculature. (c) Immunological effects, where there is a swift and significant influx of leukocytes (neutrophils, T-lymphocytes, plasma cells, macrophages, and mast cells) which may lead not only to tumor destruction but to the generation of a long-term anti-tumor immunity [73,74,75]. A major advantage of PIT in this context is its selectivity and immune cell sparing effect, which in non-targeted PDT may lead to the destruction of immune cells and compromise long-term anti-tumor immune responses [76]. Anti-tumor immune responses mediated by PDT have also been shown to be more robust in a vaccination-based model, as compared to that induced by UV or ionizing radiation [77]. This was attributed to the activation of DCs and subsequent production of IL-12, which in turn is required for the development of a Th1-based immune response [78]. Several preclinical [79,80] and clinical studies [81,82] have clearly highlighted the potential of this therapy in generating systemic anti-tumor immune responses which could greatly benefit OvCa patients where detection of peritoneal micrometastases is a major therapeutic challenge.

## 3. Photoimmunoconjugates and Photoimmunotherapy

**Figure 4:**
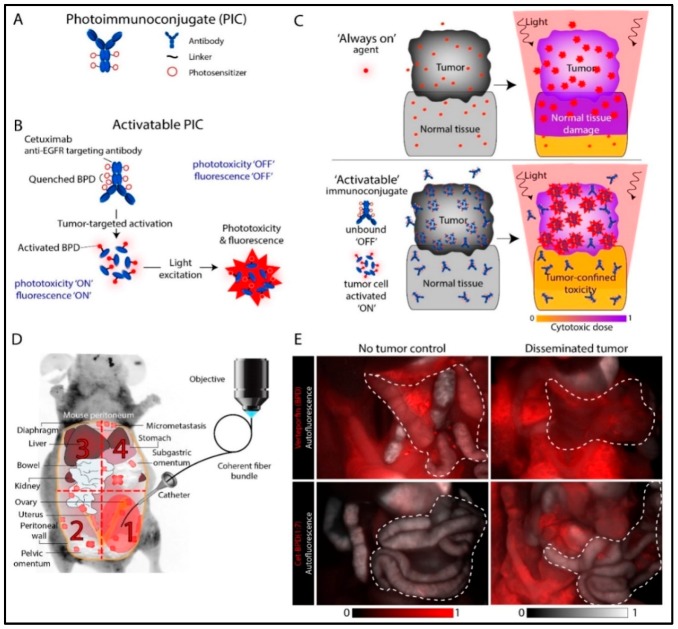
Tumor-targeted activatable photoimmunotherapy. (**A**) Pictorial representation of PICs. (**B**) Mechanism of tumor-targeted PIC activation. (**C**) The PS in PICs stay quenched under normal conditions. Once internalized in the target cells, they are dequenched through lysosomal degradation for tumor-targeted activatable photoimmunotherapy (taPIT). (**D**) Mouse model of micrometastatic OvCa and the scheme for endoscopic fluorescence imaging. (**E**) Comparison of fluorescence (red) from free benzoporphyrin derivative (BPD) (upper panel) and PIC (lower panel) administered to mice in no tumor controls (left panel) and with disseminated tumors (right panel). Adapted from Spring et al. 2014 [86].

A limitation of intraperitoneal PDT in OvCa is the lack of compounds that are both good PSs and good tumor localizers. Although initial preclinical and clinical studies with free PSs demonstrated promising results, their use was limited due to the dose-limiting damage to internal organs [9,11]. PIT has been explored to circumvent this problem by combining the tumor-localization properties of monoclonal antibodies (MAB) with phototoxic properties of PSs. PIT involves the covalent linkage of a PS to an antibody, creating a PIC. An incubation period following PIC administration allows the antibody to potentially block the targeted receptor activation, followed by uptake and processing of the PIC by the target tissue. By combining the individual modalities, the efficacy of each is improved. Erbitux, for example, recognizes and blocks the epidermal growth factor receptor (EGFR) and is FDA approved for the treatment of metastatic colorectal cancer. EGFR is a rational target because it is overexpressed in 70–90% of advanced OvCa and is associated with aggressive and resistant tumors. Interestingly, some of the early work explored the inhibition of EGFR signaling in combination with benzoporphyrin derivative (BPD)-based PDT. A synergistic response between immunotherapy and PDT was observed with increased survival in vivo [83]. This was followed by conjugation of PSs to the EGFR antibody to achieve synchronized pharmacokinetics and enhance synergistic treatment outcomes. Growth factor receptor–targeted immunotherapy is suggested to be particularly promising because it exploits blocking the dependence of tumor cells on specific molecular pathways critical for survival and growth. This is made possible by synthesizing PICs in a way that the MAB remains functional in its ability to block receptor signaling. The persistent phototoxicity of PICs due to the “always-on” nature of the PS molecules can be minimized by using tumor-targeted activatable PIT (taPIT) where PS loading on the antibody can be optimized to attain a self-quenched “off state”, which can be activated to an “on state” following target site binding, internalization and lysosomal degradation of the PICs (Figure 4A–C). This approach of lysosome activated probes has been demonstrated in various studies to enhance specificity and selectivity in therapeutic and diagnostic applications (Figure 4D,E) [84,85,86]. While no therapy can be entirely selective, use of EGFR-targeted PIC in OvCa has some significant advantages—(i) EGFR is often overexpressed in OvCa leading to highly selective PS delivery in cancer cells compared to normal tissues, (ii) greater dependence of cancer cells on EGFR signaling, and (iii) confinement of light to the peritoneal cavity [87]. 

In addition to providing an additional level of selectivity, the use of MAB-PS conjugates increases the versatility of PDT by allowing the use of compounds that are good PSs but have poor biodistribution properties on their own. MABs have been used for improved diagnosis and therapy of cancer for some time. Radiolabeled and fluorescent MABs [88,89] have been extensively used for diagnosis and image-guided surgeries [90,91]. For therapeutic applications, conjugates with radioisotopes, cytotoxic drugs, protein toxins [92], cytokines [93], and boron compounds for neutron capture [94,95] have been investigated. Besides increased selectivity, other potential advantages of PIT over conventional therapy with MAB alone or with MAB coupled to radioisotopes, drugs, or toxins are (i) since it is primarily used as a carrier, the MABs do not require any in vivo effector activity (e.g., complement fixation); (ii) in contrast to most drugs and toxins, the PS can act at the cell membrane as well as intracellularly [96]; (iii) PIT may stimulate the host immune response which may help eliminate the tumor as demonstrated by Steele et al. [97] using MAB-HP conjugates. Since the initial report by Mew et al. [98], we [14,20,86,99,100,101,102] and others [57,103,104,105,106,107,108] have demonstrated the feasibility of PICs for the preferential killing of selected cell populations in various systems [16,19,86,109,110,111]. In the 1990s, Schmidt et al. [23,24] prepared PICs of MABs recognizing CA125 on human OvCa cells and performed pilot studies of PIT in OvCa patients. While the results were encouraging, technical challenges such as uniform light delivery and toxicity remained. However, given recent advances in light delivery and dosimetry [112], antibody conjugation chemistry [113], and a deeper understanding of molecular targets, pathways, and the mechanistic interactions between PIT and conventional chemo- and radio-therapies, as described in Section 7, we believe that the PIT field has matured to a point where renewed clinical evaluation is warranted.

## 4. PIC Design Considerations

In addition to the relevant antibody targets for OvCa described below, there exist numerous aspects regarding PIC design and delivery that can be modified to enhance treatment and diagnostic efficacy of the system. Given the unique characteristics of disseminated OvCa that make effective treatment so challenging, careful consideration and optimization of PIT parameters, including the method of PS conjugation and modification of MAB biophysical properties, will be essential to improve treatment outcomes. While discussed here in the context of OvCa, the following considerations may be relevant to PIT in other diseases as well.

### 4.1. Direct vs. Indirect Conjugation

**Figure 5:**
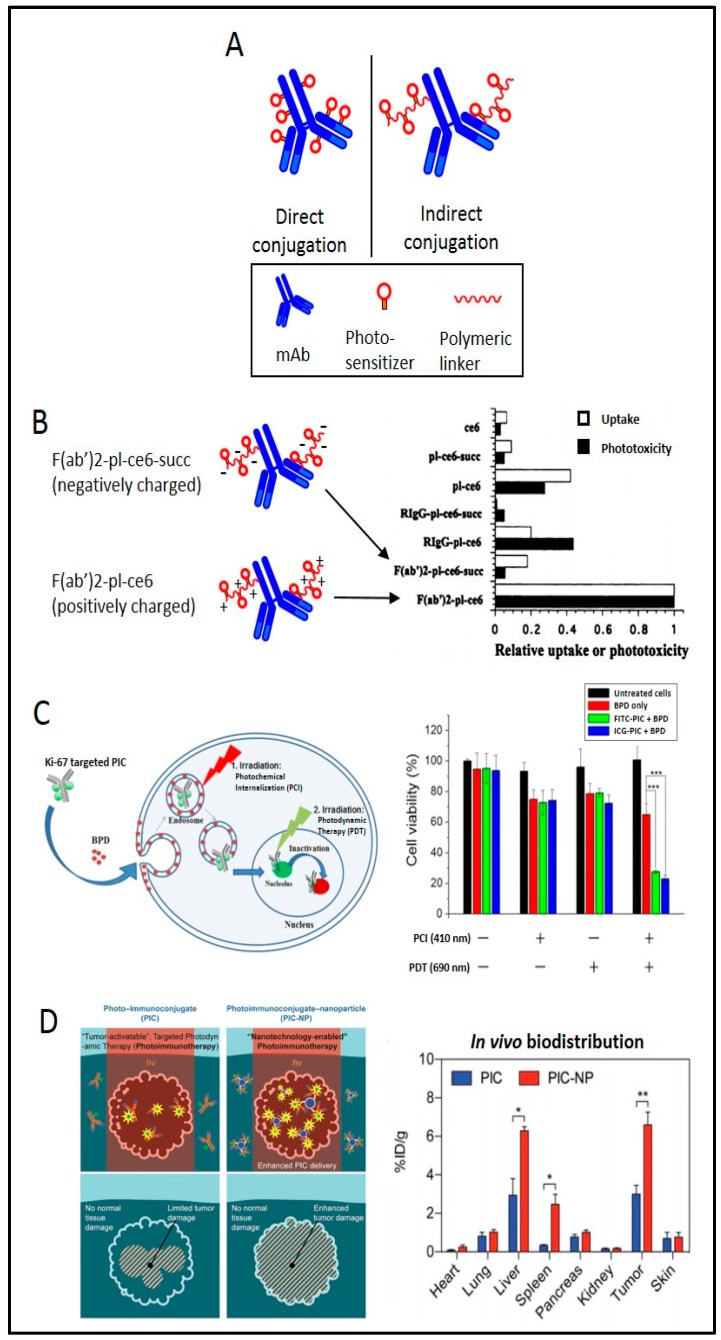
PIC design and effects. (**A**) Pictorial representation of direct vs indirect conjugation of the PSs to the antibody. The PS can be conjugated to the antibody either directly or through an intermediate polymer which increases the PS loading. (**B**) Effect of charge on cellular uptake and phototoxicity. PS conjugated to positively charged (poly-lysine conjugated) or negatively charged (succinylated poly-lysine conjugated) polymers were conjugated to the F(ab)2 fragment of OC125. The positively charged PIC led to an increase in both cellular uptake of PS and phototoxicity. Adapted from Hamblin et al. 1996 [17]. (**C**) Enhancing intracellular targeting by preventing lysosomal degradation. After co-incubation with PS (BPD) and fluorescein isothiocyanate-PIC (FITC-PIC), cells were sequentially irradiated with a low dose of 690 nm light to activate BPD-induced PCI and destabilize endosomal membranes, allowing PIC to be released into the cytosol. The second irradiation with 490 nm light initiates FITC-PIC mediated PIT to selectively kill Ki-67 expressing cells. Adapted from Wang et al. 2015 [123] and Wang et al. 2016 [108]. (**D**) Increase in BPD uptake in OvCar-5 cells due to the “carrier effect.” The binding and uptake of a single PIC results in the endocytosis of other nanoparticle-bound PICs. The “carrier effect” results in a significantly higher cellular uptake of PS and increases therapeutic outcomes in vivo. Adapted from Huang et al. 2018 [124].

A basic, yet important, consideration of PIC synthesis is how to conjugate the PS to the MAB. Synthetic strategies can be broadly separated into two methods—direct and indirect. Direct conjugation involves covalently attaching the PS onto the heavy and/or light chains of the MAB through reactive residues (typically lysine). The indirect method, which garnered significant attention in many earlier studies of PIT, involves conjugating multiple PSs onto a polymer composed of polyglutamic acid [14,20,109], poly-lysine [15,16,17,19,114], or HPMA (N-(2-Hydroxypropyl)methacrylamide) [115,116,117,118,119,120] (Figure 5A). The PS-loaded polymer chain is then attached to an OvCa antigen-targeting MAB or antibody fragment. This strategy enables loading ratios of up to 40 PS molecules per MAB while generally maintaining the antigen-binding ability of the MAB and the photophysical properties of the PS [20]. At first glance, this would appear to be a promising strategy to overcome the reduced intracellular accumulation of PSs when delivered via PIC. However, the increased complexity involved with this approach has made it difficult to reproducibly synthesize pure immunoconjugates. As a result, the majority of recent studies have involved directly conjugated PICs, as their synthesis is more straightforward and the purity and loading ratios are easier to control. Advances in polymer and conjugation chemistry and their more recent applications to targeted drug delivery systems, however, have enabled researchers to control the homogeneity, drug release properties, and subcellular localization of the payload. These improvements have been exploited in related systems with promising results [121,122]. Given that increased control over activation and subcellular localization of PSs would be a step towards addressing some of the toxicity- and efficacy-related disadvantages of PIT in OvCa. Application of these more recent conjugation strategies in this context warrants renewed exploration.

### 4.2. Charge-Based Enhancement of Cellular Uptake

Electrostatic charge is known to play an important role in the cellular uptake of proteins and nanomaterials. Given the overall negative charge of cellular membranes, modification of PS conjugates with positively charged moieties has generally been shown to non-selectively improve cellular uptake of PSs [114,125]. In a study by Hamblin and colleagues [17], this strategy was exploited to improve selective uptake and phototoxicity of chlorin-e6 in OvCar-5 cells using positively charged antibody fragments, where poly-lysine (positively charged) or succinylated poly-lysine (negatively charged) linkers carrying PSs were conjugated to the F(ab)2 fragment of OC125. The cationic PIC led to an uptake 17 times more than that of the anionic version after 24 hours incubation and enhanced phototoxicity to a similar degree in vitro (Figure 5B). A follow-up study by Molpus et al. [16] similarly found that cationic PICs were modestly more effective than anionic PICs in an orthotopic mouse model and reduced tumor burden by 90% in the short term. Both studies utilized indirect PS conjugation methods in order to manipulate the charge of the polymer and immunoconjugate as a whole. Further development of polymer-based indirect conjugation strategies will enable better control over physical and biological properties, such as charge, size, and clearance rates. Given the extensive literature on the effect of antibody charge on biodistribution and cellular uptake [126], optimization of this parameter presents an opportunity to improve the delivery of PSs and reduce dose-limiting off-target toxicities often associated with intraperitoneal PDT.

### 4.3. “Multiple Epitope-Targeting” for Enhancing Efficacy

Antibodies usually target specific epitopes on the corresponding target antigen. Attempts at enhancing antibody binding to the target antigens have involved the targeting of multiple epitopes on a single antigen in order to improve therapeutic outcomes. This strategy for PIT was investigated by Savellano et al. [127], where the administration of two separate PICs made of antibodies targeting different epitopes of the HER2 receptor were shown to be significantly more effective on a per mole PS basis than when only one epitope was targeted. This study suggests that it is possible to maintain phototoxicity while reducing the total amount of PS administered in order to minimize off-target toxicity associated with nonspecific PIC uptake. Furthermore, targeting multiple epitopes simultaneously may inhibit treatment evasion due to mutations and heterogeneity of HER2 among the cell population. However, the authors concede that the heterogeneity in expression levels of a given receptor will reduce overall efficacy and increase selection pressures on the disease as a whole. Targeting multiple receptors simultaneously through PIT may be a promising strategy to more effectively control the disease by combining the improved PS uptake demonstrated by Savellano and colleagues [127], with the synergistic effects seen in some antibody combination therapies [128].

### 4.4. Preventing Lysosomal Degradation and Enhancing Intracellular Targeting

Intracellular receptors have also been investigated as targets for PIT. However, effectively delivering PIC to intracellular compartments presents a significant challenge, as many endocytic uptake pathways result in hydrolysis of the protein and loss of targeting function. In a series of studies led by Rahmanzadeh [108,123,129], liposomes, cell-penetrating peptides, and photochemical internalization (PCI) were investigated as strategies to enhance the intracellular delivery of a Ki67-targeted PIC. In one of these studies, benzoporphyrin derivative (BPD) was utilized to initiate PCI and release of a FITC-PIC into the cytoplasm following endocytosis, as BPD has been shown to localize in the mitochondria and endosomes/lysosomes. After co-incubation with BPD and FITC-PIC, cells were irradiated with a low dose of 690 nm light to activate BPD-induced PCI and destabilize endosomal membranes to allow for PIC to be released into the cytosol. A second irradiation of 490 nm light was delivered to initiate FITC-PIC mediated PDT to selectively kill Ki-67 expressing cells. Significant cell killing was only seen upon this sequence of irradiation, indicating the promise of this type of light-activated approach to intracellular drug delivery (Figure 5C). Given the toxic side effects associated with diffuse intraperitoneal PDT, strategies that enable lower overall light doses without compromising treatment efficacy will be important in translating this technology to the clinic. Similar approaches whereby low-dose PDT is used to manipulate subcellular localization of other cytotoxic species, instead of directly killing tumor cells, represent an underexplored area of PDT. Nearly all anti-cancer agents require specific localization within cells in order to act on its target. The use of PCI and similar photodynamic strategies may enable targeting of previously inaccessible intracellular targets or antigens and allow PDT to find a unique niche in this treatment setting as a non-cytotoxic therapy.

### 4.5. PIC-Based Enhancement of Nanoparticle Uptake

The targeting properties of PIC have recently been exploited as a means to selectively deliver nanoconstructs to malignant cells. In a study by Huang et al. [124], EGFR-targeting BPD-conjugated PICs were covalently conjugated to fluorescent dye-containing poly(lactic-co-glycolic acid)–poly(ethylene glycol) (PLGA-PEG) polymeric nanoparticles. The resulting PIC-nanoparticle (PIC-NP) conjugates exhibited improved cellular uptake of BPD and phototoxicity in EGFR overexpressing OvCar-5 (OvCa) and U87 (glioma) cell lines compared to PIC. It was hypothesized that this increased uptake of BPD was due to the “carrier effect”, whereby the binding and uptake of a single PIC resulted in the endocytosis of all other nanoparticle-bound PIC into the cell (Figure 5D). These results were replicated in a subcutaneous OvCar-5 mouse model, where tumor uptake of BPD and response to PDT were enhanced in PIC-NP treated animals. Using a cetuximab-conjugated liposome, Obaid et al. show exquisite selectivity in 3D models of pancreatic cancer [130]. The liposomal targeting has the potential for delivery of a variety of cargo and it would be interesting to see if this selectivity observed in pancreatic cancer holds in OvCa models where it is absolutely necessary to protect the surrounding vital structures. This strategy using PIC as a targeting moiety could be expanded to selectively deliver other nanoconstructs, such as liposomes or gold nanoparticles. Furthermore, this platform will allow codelivery of other therapeutic agents encapsulated within the nanoparticle, such as cisplatin and paclitaxel [131]. Given the importance of sequence of delivery in PDT-based combination therapies, this platform would enable precise control over the timing of delivery and activation of the various therapeutic agents. With antibody-targeted nanoconstructs [132] and PICs [133,134] recently moving into clinical trials, further development of PIC-targeted nanoconstructs may enable PIT-based combination therapies for OvCa patients in the future. 

## 5. The Rationale to Target EGFR for OvCa Photoimmunotherapy

**Figure 6:**
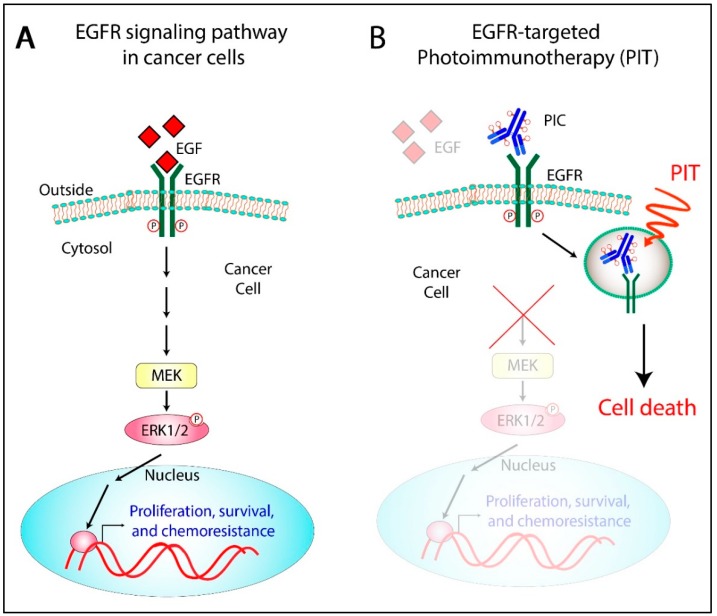
Multi-functional epidermal growth factor receptor (EGFR)-targeted PIT. (**A**) In cancer cells, overexpressed EGFRs bind to the corresponding ligands and promote cell growth, proliferation, metastasis, angiogenesis, etc. (**B**) The administration of PICs targeting EGFR leads to selective accumulation of the PS in the malignant tissue and inhibition of EGFR signaling pathway and induces localized cell death upon irradiation (right panel).

Growth factor receptors have been recognized as important targets in cancer treatment [135,136,137,138]. Amongst these, the EGFR family of receptors has attracted significant attention [139]. The ERBB family of proteins comprises four closely related receptor tyrosine kinases with structural similarity to the epidermal growth factor receptor (EGFR). The four members are human epidermal growth factor receptor 1 (HER1; ERBB1 also called EGFR), HER2 (ERBB2), HER3 (ERBB3), and HER4 (ERBB4) [140]. Upon ligand binding, EGFR elicits cellular responses through multiple divergent pathways. These pathways control a number of cellular processes, such as growth, motility, and production of growth factors [141]. The ectodomain of the receptor contains ligand-binding sites, while the protein-kinase catalytic sites are in the intracellular domains. Receptor signaling follows five major pathways *viz.* 1) The Ras/Raf/MEK/Erk pathway, 2) STAT pathway, 3) PI3K/AKT pathway, 4) Src kinase pathway, and 5) PLCγ/PKC pathway [142] (Figure 6). While the Ras/Raf/MEK/Erk and STAT pathways are involved in cellular differentiation and proliferation, the PI3K/AKT and PLCγ/PKC pathways are important for cell survival and motility, respectively. 

Under normal conditions, the expression of EGFR in the epithelial lining of ovarian tissue is generally low. However, its overexpression has been reported in 30–98% of OvCa cases [143] and is thus considered a strong prognostic indicator for OvCa. It binds to various ligands, including EGF and TGF alpha, and contributes to the active malignancy of OvCa by promoting cell growth, cell migration, angiogenesis, and conferring resistance to apoptosis.

**Figure 7:**
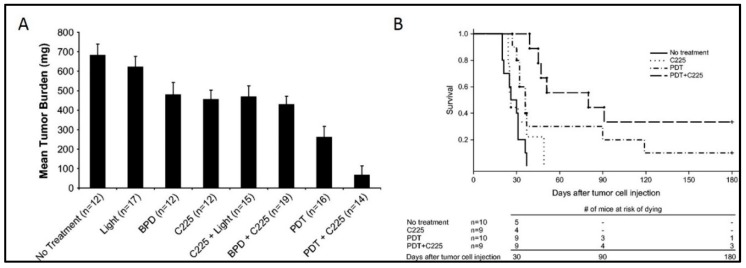
(**A**) Mean tumor burden for mice treated with either C225 or PDT monotherapy, compared with a combination therapy of C225 and PDT. (**B**) Kaplan–Meier survival curves for mice treated with photodynamic therapy only, C225 only, and mice treated with a combination therapy of PDT and C225. Combination treatment with PDT and C225 resulted in a significant enhancement in survival as compared to the individual monotherapies. BPD = benzoporphyrin derivative. PDT = photodynamic therapy. C225 (Cetuximab, Anti-EGFR antibody). Adapted from del Carmen et al. 2005 [83].

Overexpression of EGFR is an attractive and reasonable target for OvCa management; therefore, many antibodies targeting EGFR have been developed and are in clinical use. These include cetuximab, panitumumab, and necitumumab which act by competing with the ligand-binding sites on the extracellular domains of EGFR and inhibiting downstream signaling pathways. Previous studies from our group and others have demonstrated improved therapeutic outcomes with the combination of EGFR inhibition and PDT as compared to monotherapy [83]. The study by Del Carmen et al. showed a synergistic enhancement of tumor control (Figure 7A), increased survival, and a 33% cure (Figure 7B) in mice with disseminated disease treated with both modalities. Conjugation of PS to therapeutic EGFR antibody (Cetuximab), as in PICs, therefore provides a combination therapy with a single therapeutic agent along with a targeting specificity which may overcome the limitations in previous clinical studies [9,11,144]. Although PDT has been shown to degrade EGFR [145,146], it also sensitizes cells to EGFR-based inhibitors, thus highlighting the potential of this combinatorial approach. Given the selectivity afforded by the EGFR-based targeting and the simultaneous inhibition of the EGFR-based survival signaling pathways, PIT with EGFR targeting holds great clinical potential where phototoxicity due to non-specific PS distribution has been a limiting factor. Most studies related to PIT of OvCa have been performed with EGFR antibodies, discussed in detail in Section 3 and Section 4. Other molecular targets that have been explored in this disease context are discussed in the following section.

## 6. Other Molecular Targets for OvCa Photoimmunotherapy

EGFR is the most studied target for PIT of OvCa mainly due to its overexpression and localization on the cell surface. However, certain mutations in the EGFR gene lead to a reduction in anti-EGFR antibody (Cetuximab) binding and therapeutic efficacy. Although this resistance can be overcome by utilizing antibodies directed against different epitopes (Panitumumab) [147], other molecular targets for OvCa have also been identified and exploited for their therapeutic potential. While most of these molecular targets are well-established markers for diagnostic applications, they nevertheless provide an alternative for targeted OvCa therapies. Of the various molecular targets identified for OvCa, Ki67, HER2, Folate receptor α (FRα) and Muc16 have been reported for PIT as well and are discussed below.

### 6.1. Human Epidermal Growth Factor Receptor 2 (HER2)

In a study by Sato et al. (2014), IRDye-700DX-conjugated anti-HER2 antibody Trastuzumab was used to perform NIR-PIT in 3D OvCa spheroids with repeated irradiation, which resulted in complete cell killing and was effective in reducing tumor volumes in vivo in both subcutaneous flank and disseminated peritoneal models of OvCa [51].

### 6.2. Ki67

Ki67 is a nuclear protein closely related to cell division. Its expression and localization are indicative of the proliferation status of cells. For this reason, it has been widely used as a prognostic marker for different cancer types and for categorizing cancers into different grades based on the proliferation status. Although the prognostic significance of Ki67 expression has not been established in the case of OvCa, it is generally accepted that high Ki67 expression is indicative of aggressive tumors and poor prognosis. Under normal conditions, a minimal expression of Ki67 is observed and a decrease in the Ki67 levels indicates an exit from the cell cycle (G0 phase). However, stimulation with growth factors leads to an increase in Ki67 expression and entry of cells into the actively dividing state (S phase). Inhibition of Ki67 with either siRNA or antibodies has been shown to reduce cell division rates, highlighting its role as a target molecule in cancer therapeutics [148].

In an interesting study, Rahmanzadeh et al. have reported the encapsulation of a FITC-conjugated Ki67 antibody in liposomes for nuclear delivery [129]. The photo-immunoconjugate-encapsulating liposomes (PICELs) were shown to enter OVCAR cells in both 2D and 3D cell cultures. Upon cellular uptake, the released antibody localized in the nucleolus and inactivated Ki67 upon irradiation. The chromophore-assisted light inactivation (CALI) [149] observed in this study is amongst the first few reports on targeted inactivation of Ki67 for achieving therapeutic benefits [129,150]. Importantly, the inactivation of Ki67 was observed only when the antibody TuBB-9 (targeting a functionally active form of Ki67) was used and was not observed in case of MIB-1 antibody (targeting a different epitope), suggesting the importance of targeting the active Ki67 fraction (involved in rRNA synthesis) [151]. A significant decrease in viability was observed in both 2D and 3D OVCAR-5 cell cultures but not on the fibroblast cell line (MRC5), with low proliferation rates. The cell death observed in 3D OVCAR-5 cultures was slightly lower than that observed in 2D OVCAR-5, likely due to the differences in the Ki67 expression patterns arising due to the culture geometries [152]. The mechanism of CALI for Ki67 involves crosslinking and inactivation of the protein leading to an inhibition of transcription and cell death. 

Although the prognostic significance of Ki67 is well established in determining cancer stage and aggressiveness in general, its targeting for achieving a therapeutically relevant outcome is yet to be explored. Targeting and inactivation of intracellular targets remain a major challenge. As demonstrated in the study by Rahmanzadeh et al. [129] and others [153,154], this could be achieved by delivering PICs intracellularly through nanoconstructs or PCI (as discussed in Section 4.4). As patients with high Ki67 expression respond better to first-line chemotherapy treatments [155], the targeted inactivation of Ki67 provides an alternative option for diagnostic and therapeutic applications.

### 6.3. MUC16

MUC16, a member of the membrane-associated mucin (MAM) family of proteins, is expressed on the apical membranes of many epithelial surfaces such as the cornea, conjunctiva, respiratory tract, female reproductive tract, etc. It primarily functions to serve as a wetting agent for the hydration/lubrication of the epithelial surfaces and formation of a protective barrier. In addition, many mucins including MUC16 have been shown to bind cytoskeletal elements through their intracellular cytoplasmic domains and enhance cell proliferation and differentiation through a cleavable cytoplasmic domain [156]. Some mucins are also known to associate with surface receptors *viz.* ERBB2 and enhance cellular proliferation and differentiation [157]. An EGF dependent phosphorylation of the cytoplasmic tail of MUC16 has been reported upon exposure to EGF, suggesting a potential role of MUC16 in EGF signaling [158]. Other studies have established the promotion of cancer cell motility, metastasis, and tumorigenicity through the cytoplasmic domain of MUC16 [159]. MUC16 overexpression has been reported in human OvCa, where it plays an important role in tumor proliferation, drug resistance, and immune evasion. For this reason, OC125, a MUC16-targeting antibody, has been reported for the diagnosis and treatment of OvCa [160].

In a series of studies, Goff et al. reported the development of chlorin-e6 conjugated OC125 antibody for use in OvCa. The PS was conjugated to polyglutamate prior to antibody conjugation to increase PS uptake in the target cells. To maintain antibody specificity, conjugation was performed using the carbohydrate moiety at the hinge region of the antibody placing the PS away from the antigen-binding site and thereby improving the molecular specificity of the conjugate [20,101]. While the binding of PSs to polymers (polyglutamate) has been shown to increase photochemical efficiency, separate studies have also demonstrated increased phototoxicity for neutral and anionic PICs as compared to cationic PICs [125]. The photo-immunoconjugates (PIC) developed in these studies were shown to be specific against human ovarian tumor ascites. The PICs showed tumor accumulation in vivo in ascitic ovarian tumor models [14], with a significant reduction in tumor cells upon PDT. At high PS concentrations and irradiation doses, a single PDT regimen drastically decreased the tumor viability, but secondary lethal toxic effects were also observed. However, multiple low doses were not only able to decrease tumor cell viability, but led to a decrease in toxicity and morbidity with a significant increase in survival [109].

MUC16 expression is considered to be a diagnostic marker for OvCa. The ectodomain of MUC16 can be cleaved and released in circulation, the detection of which in serum is frequently performed for tumor diagnosis. However, a correlation between MUC16 expression and resistance to chemotherapy has not been established. A major drawback associated with MUC16 targeted immunotherapies has been the binding of the therapeutic antibodies to free MUC16 proteins in circulation, leading to the reduction in the effective therapeutic dose. The inhibition of MUC16 binding to mesothelin (an interacting partner of MUC16) has also been unsuccessful for the same reasons [161]. Although the potential of MUC16 has been demonstrated in many studies, a better understanding of the physiology of MUC16 expression and release into the circulation may help in designing improved therapeutic alternatives. 

### 6.4. Folate Receptor α (FRα)

Although not PIT, folate conjugated photosensitizers have also been explored for targeting OvCa. Folate receptor α (FRα) is frequently overexpressed in OvCa and high FRα expression is associated with poor prognosis in OvCa patients. It has also been shown to confer resistance to chemotherapy by decreasing the expression of Bax and increasing the expression of Bcl2, which interestingly is a direct target of PDT [162]. Recent reports from Sergey Mordon’s group have demonstrated the selectivity and specificity of folate-conjugated photosensitizers in targeting metastatic ovarian tumors. Moreover, the high expression of FRα in OvCa and its relatively low expression in the peritoneum, intestines, and kidneys suggest a potential application in OvCa management [163,164,165]. It would be interesting to have a comparison of this work with antibody targeting of the folate receptor, although, to the best of our knowledge, this has not been reported.

## 7. PIC-Based Combination Therapies

While it has shown considerable promise as a standalone therapy in OvCa, preclinical evidence suggests that PIT is most likely to be effective in combination with other cytotoxic therapies. PIT itself is inherently a combination therapy, as it is composed of a receptor blocking antibody and a phototoxic sensitizer. The therapeutic interaction of these two core components of PIT was examined by del Carmen and colleagues (del Carmen-Hasan 2005) in which anti-EGFR antibody was combined with untargeted PDT in an orthotopic xenograft mouse model. The combination significantly reduced tumor burden and increased animal survival compared to either therapy alone, providing a strong therapeutic rationale for combining PDT and immunotherapy in addition to the inherent selectivity of PIT. PIT has been shown to act synergistically with chemotherapy as well. Both untargeted and targeted forms of the cytotoxic drug SOS thiophene and the PS mesochlorin-e6 were demonstrated to be highly synergistic at low doses by Hongrapipat et al. [115], suggesting that PIT-based combinations may enable dose reductions of toxic chemotherapies while maintaining efficacy in this highly resistant disease. This effect was confirmed in vivo by Rizvi and colleagues [131], whereby cisplatin followed by PIT demonstrated comparable or better antitumor effects than 2 cycles of chemotherapy. Additionally, numerous studies have shown that PDT synergizes with various chemo- and radio-therapies in a range of cancer types [41,166], indicating that this is a fairly generalizable phenomenon.

The resistance to radio- and chemo-therapy agents frequently observed in recurrent OvCa leaves this patient population with few or no treatment options. In this context, PIT makes a compelling case to improve survival outlooks for this disease. Studies by Goff et al. [20] and Duska et al. [15] investigated the effects of combined PIT and cisplatin treatment on ex vivo samples from OvCa patients. In both studies, patient tissues resistant to standard chemo- and radio-therapy were demonstrated to be responsive to PIT. In the study by Duska et al., platinum treatment following PIT was demonstrated to have an additive therapeutic effect in cisplatin sensitive cancer cells, while, remarkably, a strong synergistic effect was observed in platinum-resistant cells, suggesting that PIT can resensitize these tumors to chemotherapy (Figure 3). These data suggest that the use of PIT fills in a crucial niche in the treatment of OvCa, where resistance to chemo- or radio-therapy is a major problem. These types of PIT-based resensitization strategies may hold promise for increasing treatment options in these patients. 

The synergistic and re-sensitization effects observed in PIT-based combination therapies such as these may represent a new strategy for managing patients with advanced disease. Typical treatment regimens involve administering the maximum tolerated dose of toxic chemo- or radio-therapies with the hope of eliminating any remaining cancer cells. However, this approach comes with dangerous and often dose-limiting side effects, negatively impacting patient quality of life and limiting these treatments only to those with favorable health status. The non-overlapping toxicity profiles of PIT and chemotherapy, and the ability to reduce the dose of these cytotoxic drugs following PIT-based “priming” of the disease may open up new treatment options for patients who may otherwise not be eligible or responsive to classical therapeutic regimens. Furthermore, optimized PIT regimens that enable dose de-escalation of toxic chemotherapies while maintaining antitumor efficacy hold promise as a means for improving patient quality of life and more humanely treating this devastating disease. 

## 8. Conclusions and Future Directions

Current treatment methods for OvCa involve surgical debulking in combination with chemo- and radio-therapy. However, the disseminated nature of epithelial OvCa (EOC) poses a major challenge during therapeutic interventions. Targeted therapies using antibodies (Bevacizumab, Cetuximab, etc.) have therefore been used for the treatment of EOCs. PDT has been shown to be effective against chemoresistant disease. It has also been reported to synergize with and re-sensitize resistant disease to chemotherapy; however, diffuse PDT in the anatomically complex peritoneal cavity often results in significant toxicity to the surrounding healthy tissue. PIT compensates for this potential for collateral damage through antibody-mediated tumor-specific accumulation of PSs and spatiotemporal control of light irradiation and PS activation. Perhaps the shortcomings noted in the previous clinical trials with non-specific phototoxicity and bowel toxicity could have been avoided with PIT [23,24]. Clinical trials with PICs composed of IRDye700DX with cetuximab (RM-1929; Phase I and II; NCT02422979 and ASP-1929; Phase III; NCT03769506) are underway, not for OvCa, but for recurrent head and neck cancer treatment [133,134], and may provide further insights into the utility of PIT.

Although PDT and PIT have demonstrated great potential for cancer treatment, there are limitations that prevent the use of PIT as a standard of care for cancer treatment in general and OvCa in particular. While delivery of PSs through PICs improves non-specific toxicity and tumor localization, the effective concentration of PS actually delivered to the target cells is significantly reduced when compared to free PS administration [14,20]. However, PICs constructed with nanoparticles and liposomes allow for a higher payload of photosensitizers, as reported by our group [124,130]. 

Advancements in endoscopic methods of light delivery further add to the ease of incorporating PIT/PDT into the workflow for the management of advanced OvCa. Limitations in the penetration depths of light has for the most part been addressed by the use of optical fibers that are able to access different parts of the body. More forward-looking options include the use of bioluminescent molecules co-delivered with PSs. These systems rely on the bioluminescence resonance energy transfer (BRET) where the bioluminescent agent (protein, quantum dots, etc.), when co-delivered with PSs, may excite the PS resulting in the desired photochemical reactions [167]. Although in its infancy, bioluminescent PDT (BL-PDT) has been reported to treat tumors in pre-clinical models. Other approaches used to circumvent the short-comings of limited light penetration are X-ray based PDT [168,169] and the use of upconversion nanoparticles [170].

In summary, recent chemical and technological advances place PDT/PIT in the right place and the right time as a complementary therapy for OvCa. It is effective against chemo- and radio-resistant diseases, targetable by methods currently in use for conventional treatments, and can be easily incorporated into the traditional workflow for mopping-up residual disease and salvage therapy. It therefore has the potential to fill a niche for resistant residual disease management for which there are currently no other effective options. 

## Abbreviations

BPD:	benzoporphyrin derivative
EGFR:	epidermal growth factor receptor
EOC:	epithelial ovarian cancer
ER:	endoplasmic reticulum
FDA:	Food and Drug Administration
FITC:	fluorescein isothiocyanate
GC:	Golgi complex
i.p.:	intraperitoneal
MAB:	monoclonal antibody
MDR:	multi-drug resistance
OvCa:	ovarian cancer
PCI:	photochemical internalization
PDT:	photodynamic therapy
PI3K:	phosphatidylinositol-3-kinase
PIC:	photoimmunoconjugate
PIT:	photoimmunotherapy
PF:	photofrin
PK:	pharmacokinetics
PS:	photosensitizers
VEGF:	vascular endothelial growth factor
